# Intracranial Somatosensory Responses with Direct Spinal Cord Stimulation in Anesthetized Sheep

**DOI:** 10.1371/journal.pone.0056266

**Published:** 2013-02-15

**Authors:** Oliver E. Flouty, Hiroyuki Oya, Hiroto Kawasaki, Chandan G. Reddy, Douglas C. Fredericks, Katherine N. Gibson-Corley, Nicholas D. Jeffery, George T. Gillies, Matthew A. Howard

**Affiliations:** 1 Department of Neurosurgery, University of Iowa Hospitals and Clinics, Iowa City, Iowa, United States of America; 2 Department of Orthopaedic Surgery, University of Iowa Hospitals and Clinics, Iowa City, Iowa, United States of America; 3 Department of Pathology, University of Iowa Hospitals and Clinics, Iowa City, Iowa, United States of America; 4 Department of Veterinary Clinical Sciences, Iowa State University, Ames, Iowa, United States of America; 5 Department of Mechanical and Aerospace Engineering, University of Virginia, Charlottesville, Virginia, United States of America; Universität München, Germany

## Abstract

The efficacy of spinal cord stimulators is dependent on the ability of the device to functionally activate targeted structures within the spinal cord, while avoiding activation of near-by non-targeted structures. In theory, these objectives can best be achieved by delivering electrical stimuli directly to the surface of the spinal cord. The current experiments were performed to study the influence of different stimulating electrode positions on patterns of spinal cord electrophysiological activation. A custom-designed spinal cord neurostimulator was used to investigate the effects of lead position and stimulus amplitude on cortical electrophysiological responses to spinal cord stimulation. Brain recordings were obtained from subdural grids placed in four adult sheep. We systematically varied the position of the stimulating lead relative to the spinal cord and the voltage delivered by the device at each position, and then examined how these variables influenced cortical responses. A clear relationship was observed between voltage and electrode position, and the magnitude of high gamma-band oscillations. Direct stimulation of the dorsal column contralateral to the grid required the lowest voltage to evoke brain responses to spinal cord stimulation. Given the lower voltage thresholds associated with direct stimulation of the dorsal column, and its possible impact on the therapeutic window, this intradural modality may have particular clinical advantages over standard epidural techniques now in routine use.

## Introduction

Two years after Melzak and Wall introduced the gate theory of pain [1] the first use of spinal cord (SC) stimulation (SCS) in a patient with intractable pain was reported by Dr. C. Norman Shealy of the Gunderson Clinic [2]. Since that time, SCS has been employed not only for pain relief but in a broad range of other clinical applications, even though our understanding of the underlying therapeutic mechanism remains incomplete. The stimulator implantation procedure involves placing an electrode array into the dorsal epidural space overlying the SC. Complications that can sometimes necessitate revision or even explantation include: wound infections, CSF leak, pain and discomfort at the pulse generator site, lead migration, malfunction of the pulse generator, insufficient pain relief, and in one reported case, seizures [3]. Patients selected for SCS placement have failed, or are unsuitable candidates for alternative medical and surgical therapies. The proposed mechanism of action is believed to stem from activation of large diameter afferents in the dorsal columns or dorsal rootlets followed by inhibition of nociceptive small diameter fiber transmission [4]. According to the gate theory of pain, by selectively activating large diameter dorsal column fibers, nociceptive information at a segmental level is suppressed. There is also evidence that cerebral activity is altered when the spinal cord is electrically stimulated [5], in such a way as to cause decreased sensory perception and a diminished emotional response to pain [6], [7]. Certain of those studies [5], [7] employed PET and other functional imaging methods to investigate alterations in BOLD response in the thalamus, posterior parietal, anterior cingulate, as well as prefrontal cortex during spinal cord stimulation. Also, while some studies showed favorable results of SCS in failed back surgery syndrome [8], other studies found no such evidence for greater effectiveness when compared to alternative treatment [3]. With current epidural stimulators, 1–2 year sustained symptom relief has been reported to occur in 25–50% of implanted patients [9], [10], and the potential causes for this marked variability in outcomes have been examined extensively.

One major concern associated with failure of these devices to provide sustained pain relief relates to changes in stimulator lead position and electrical shunting effects due to the relatively high electrical conductivity of the cerebrospinal fluid (CSF). All existing stimulators apply electric fields across the epidural space, resulting in very limited ability to precisely trigger specific neuronal pathways within the spinal cord. For instance, it is estimated that more than 90% of the injected current is shunted away from the targeted neural tissue by the CSF. The remaining 10% (or less) reaches only the superficial 200 to 250 µm layer of the dorsal columns [11] leaving deeper axons unaffected by the stimulus. As a result, generating optimal current density distributions within the targeted populations of somatotopically organized dorsal column axons is extremely difficult and in some instances impossible via the epidural stimulation approach. If the applied voltage is too high, current may also be delivered to neighboring non-targeted structures where it can stimulate aversive pain pathways as well as other unrelated sensory fascicles thus causing significant discomfort and paresthesias. The therapeutic window, i.e., the stimulus intensity range between onset of therapeutic effect and the threshold of discomfort, is thus very limited.

We are developing a novel device capable of overcoming this fundamental limitation of existing SC stimulators: their restricted capacity to selectively activate targeted axons within the SC. Our new concept makes it possible to position the stimulating electrodes on the SC pial surface, thus directly stimulating the dorsal column bundles while minimizing leakage currents to neighboring neural structures involved in pain transmission, such as the dorsal rootlets. As a result, the therapeutic window becomes much wider and, in principle, this allows us to activate increasing numbers of targeted large diameter fibers while minimizing unintentional activation of adjacent non-targeted neural structures. Using the equipment currently under development, this approach will also make it possible for the first time to obtain chronic *in vivo* electrophysiological recordings from the human spinal cord. A description of this concept from the general medical physics perspective is available elsewhere [12], as are details of some of the preclinical testing protocols employed to date [13], [14], [15], including the fixation techniques used to position and stabilize this device [16], termed the Iowa-Patch™ (or I-Patch), on the surface of the SC in an *in vivo* ovine model.

In what follows, we present data from sheep experiments where cortical somatosensory evoked potentials (SSEP) were recorded during direct electrical stimulation of the dorsal columns carried out using a custom built neurostimulator. The aims of this systematic experiment are to objectively measure the physiological performance differences between epidural and intradural stimulation, to investigate the importance of lead positioning relative to the SC, and to determine the optimal voltage thresholds triggering robust SSEPs when stimuli are delivered directly to the surface of the spinal cord. We hypothesize that intradural direct spinal cord stimulation evokes a stronger cortical response at a lower stimulation voltage when compared to epidural stimulation. To further examine the importance of lead positioning relative to targeted spinal cord pathways, we also tested the hypothesis that stimulating the SC over the dorsal columns contralateral to the recording grid elicits a stronger evoked response and high gamma waveforms at lower voltage costs when compared to midline or ipsilateral SC stimulation. Findings consistent with this hypothesis provide objective evidence of the selective targeting capabilities of the direct spinal cord stimulation strategy.

## Materials and Methods

### Ethics Statement

Four adult sheep (58–71 kg) were used in this study, which was approved by the University of Iowa Animal Care and Use Committee (IACUC# 0902039).

### Anesthesia Protocol

**Figure 1:**
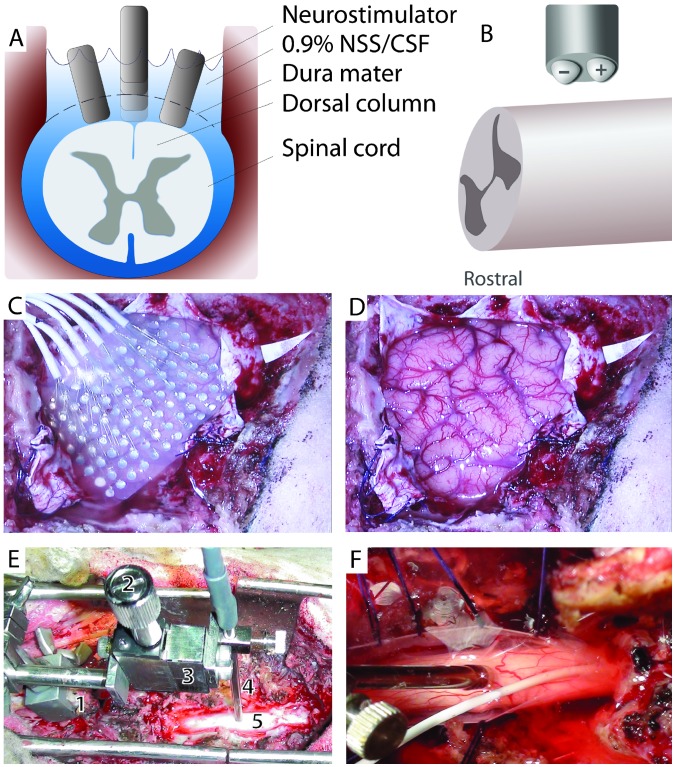
Experimental arrangement. A) Axial view of the spinal cord illustrating the different stimulator positions (intradural, epidural, midline, and lateral). B) Schematic depiction of the orientation of the bipolar stimulating electrode contacts relative to the spinal cord. The contacts were oriented in parallel with the long axis of the spinal cord, with the cathode contact being rostral to the anode contact. C and D) Photographs of the sheep’s brain before and after removal of the 96 contact subdural grid (Sheep 3). E) Photograph of the spinal cord surgical field. A mounting arm (1) is attached to the spinous process and connected to the micromanipulator (3) that is adjusted via the control (2). With this device the location of the neurostimulator (4) can be varied as needed over the mid-line of the exposed dorsal spinal cord (5) and maintained in a stable position. F) A close-up view of the neurostimulator placed in contact with the pial surface at the spinal cord midline (the dura is incised and sutured open). The field is flooded with 0.9% normal saline (NSS). In this experiment, a four contact subdural strip was also carefully inserted rostrally within the subdural space to record spinal cord potentials. The leads for the subdural strip are housed inside the white cable seen running through the lower center of the photo.

Induction of anesthesia was established with inhaled Isoflurane and maintained throughout the surgical procedure. Local anesthetic (2% Lidocaine) was injected locally prior to every skin incision. A right sided craniectomy was performed and the dura was opened. Next, a 96 contact subdural grid (Ad-Tech Medical Instruments, Racine, Wisconsin, USA) with 3.0 mm inter-contact spacing was implanted on the exposed right cerebral hemisphere of two of the sheep, while a 60 contact subdural grid with the same spacing was placed on the right hemisphere of the other two sheep. The disc-shaped electrodes were composed of a platinum-iridium alloy and were 1.4 mm in diameter. A reference electrode (a four-contact strip electrode, platinum-iridium, exposure area is 2.3 mm in diameter) was inserted in the subgaleal space over the frontal bone. Following grid placement, a multilevel laminectomy extending from T7 to T10 was performed to expose the dorsal surface of the SC. A custom-built neurostimulating probe was fixed over the intact spinous process of the eleventh thoracic vertebra using a specially fabricated micro-manipulator that was able to hold the neurostimulator precisely in place in relation to the spinal cord. This insured stable positioning of the probe throughout the duration of an experiment. Hemostasis was achieved and the field was flooded with medical grade normal saline. Thirty minutes before experimentation started, anesthesia was switched to Propofol IV infusion at a rate of 0.4 mg/kg/hr and Isoflurane was discontinued. Corneal reflex was tested periodically to ensure proper depth of anesthesia. Body temperature was maintained within normal limits using a heating pad and warm intravenous saline infusions as needed. Blood pressure, heart rate, exhaled Isoflurane concentration, and pulse oximetry were monitored and documented continuously throughout the experiments. See Figure 1 for photographs of the experimental arrangements.

### Electrical Stimulation and Data Acquisition

Using an operating microscope for direct visualization, the custom-built neurostimulating probe (impedance: 10–11 kΩ at 20 Hz) was placed on the pial surface of the spinal cord and held in position using a micro-manipulator. Constant voltage mono-phasic square pulses of 0.2 ms were applied using an IZ-2 stimulator (Tucker-Davis Technologies, Alachua, FL). The pulse delivery was in a bipolar-configuration, with the cathode placed rostrally along the long axis of the SC. The probe has two spherical contacts, each with a diameter of 1.0 mm and an intercontact distance of 2.0 mm. Electrical stimulation of the SC was performed as a function of voltage and as a function of distance from the surface of the SC, and at 2 mm lateral distances from the midline of the cord (left and right from the midline) while cortical local field potentials (LFP) were recorded from the grid. In addition, two of the four sheep underwent stimulation with the dura intact and again after a durotomy was performed. The purpose of this portion of the experiment was to study any effects that having the spinal dura mater interposed between the stimulating electrode and the spinal cord might have on the SSEPs produced during dorsal column stimulation. On average, 75±29 (STD) excitation blocks were carried out per sheep with an average of 13 hours of experimentation within the total surgical time per animal. Each block consisted of 80 stimulus presentations with an interstimulus interval (ISI) of 1.4 ms. Electrocorticography (ECoG) signals were acquired using a multi-channel neurophysiology workstation (RZ-2 and PZ-2, Tucker-Davis Technologies, Alachua, FL) with a sampling frequency of 4.88 kHz). When the experiments were completed, the animals were euthanized by administration of pentobarbital (120 mg/kg, IV).

### Signal Processing

The ECoG data was stored for offline analysis using custom-written Matlab algorithms. Power line noise at the 60 Hz fundamental frequency and its harmonics was removed using a narrow-band adaptive notch filter (59.5 to 60.5 Hz). Trials with samples exceeding 10 standard deviations away from the block mean were rejected. Average evoked potentials were computed by calculating the time series mean from 50 ms before to 200 ms after stimulus onset. Time-frequency analysis was implemented using two methods on the time series data from 500 ms before to 1000 ms after stimulus onset. The first method employed Multitaper Spectral Analysis [17] with three tapers (100 ms windows, 10 ms overlap; time bandwidth: 2). The second method computed the time-varying high-gamma band responses using a 70 to 150 Hz zero-group delay FIR bandpass filter (Matlab’s filtfilt function: order  = 900, −6 dB roll-off). By computing the Hilbert transform of the band-pass time-series, the complex analytic signal was derived. The instantaneous amplitude (high-gamma-band envelope) was computed by calculating the modulus of the analytic signal. After obtaining the instantaneous amplitude of the high-gamma band time series for every individual trial, averaging in the time domain was subsequently performed and a single averaged high-gamma band envelope was obtained for every block. High-gamma band response was then determined by calculating the peak amplitude of the averaged high-gamma band envelope occurring within the first 100 ms after the onset of the SC stimulus [18].

Lastly, we examined the effects of the voltage (0.05–18 V) and location (distance from the pial surface of the SC and laterality) of the stimulating contacts on the properties of the SSEPs. The voltage thresholds required to evoke a high-gamma band response were determined by computing the inter-quartile range (IQR) of a 200 ms segment preceding the onset of the SC stimulus. The voltage threshold, V_t_, was defined as
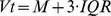
where M is the median voltage value of the prestimulus period. Any block that had 20 ms of poststimulus-period high-gamma band response exceeding V_t_ was considered to have exceeded the voltage threshold. We estimated 95% confidence intervals of the means using the following expression



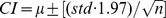
where CI is the confidence interval, μ is the mean of the samples, std is the standard deviation and n is the number of samples. The resulting values for CI are reported in the next section.

## Results

**Figure 2:**
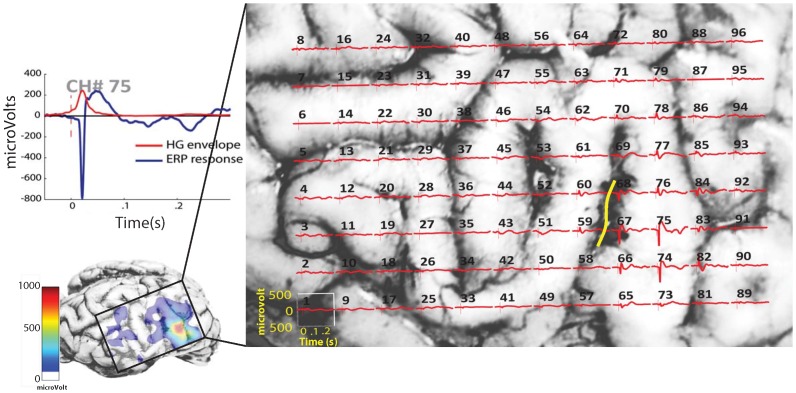
Post-mortem image of the brain of Sheep 3 with superimposed event-related potentials (ERP) in response to 5 V epidural spinal cord stimulation. The detailed time course of the ERP waveform (blue trace) and high-gamma band (HG) envelope (red trace) of a representative channel (# 75) is shown in A. Evoked response peak-to-trough magnitude as a color code (color bar) interpolated over the subdural grid contact area (cubic interpolation) is shown in B. An example of phase reversals of the evoked responses recorded from adjacent contacts is shown in the region depicted by the yellow line (C).

Local field potentials where recorded from a right subdural grid of four sheep undergoing SC stimulation. A total of 376 blocks were divided among the four sheep. The stimulus parameters were chosen based on the findings of previous pilot studies [16], [19]. An example of the evoked potential distribution over the brain is shown in Figure 2. Initial SSEP negative peak latency is 21 ms which coincides with the high-gamma band peak.

Sheep 1: The first animal underwent dorsal column stimulation both with the dura intact and post durotomy. Epidural stimulation was performed with the stimulator secured at 7 different distances from the spinal cord dura at the midline (0, 1, 2, 3, 4, 5, and 10 mm away from the dura) and 15 different voltages per distance (0.4, 0.8, 1.2, 1.6, 2, 3, 4, 5, 6, 7, 8, 9, 10, 14, and 18 V). The total number of epidural stimulation blocks was 112, each taking two minutes to complete. Once the epidural series was completed, the dura was opened and the stimulator was placed gently on the exposed dorsal pial surface of the spinal cord. Intradural stimulation was performed at 6 different distances from the midline dura (0, 1, 2, 3, 4, and 5 mm away from the SC) as well 10 voltages per distance: 0.4, 0.8, 1.2, 1.6, 2, 3, 4, 6, 8, and 10 V. Off-midline stimulation was also performed by carefully placing the stimulator on the pial surface (i.e., 0 mm away from the SC) 2 mm to the left as well as 2 mm to the right of the dorsal median septum (midline) using the same stimulation voltage protocol. The total number of intradural stimulation blocks was 80, each taking two minutes to complete.

**Figure 3:**
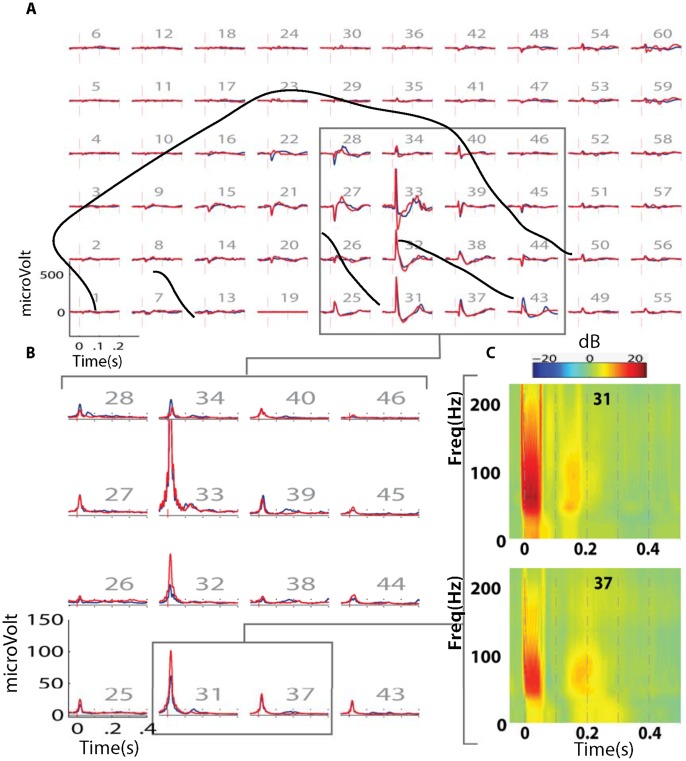
Sheep 1: Event related cortical oscillations. A) Average evoked potential distribution for epidural (blue) and intradural (red) spinal cord stimulation at 0 mm with 5 volts. Major cortical sulci are denoted by dotted lines. B) Time-varying high-gamma band (75–150 Hz) envelope for epidural (blue) and intradural (red) spinal cord stimulation in selected channels. C) Time-Frequency analysis of two representative channels during epidural stimulation. The y-axis denotes frequency in hertz and the x axis denotes time in seconds centered at stimulus onset. The color scale represents relative power change with respect to pre-stimulus values in decibels (dB).

**Figure 4:**
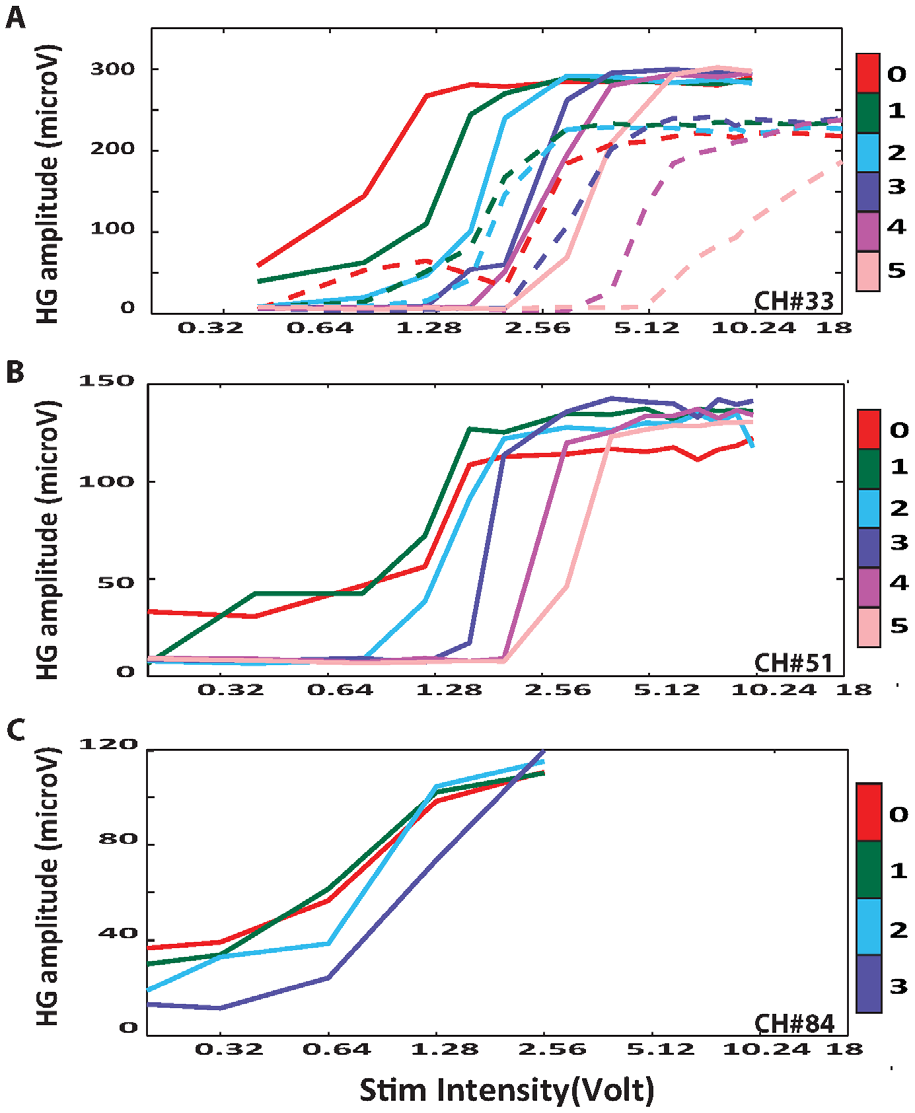
Distance-Voltage-Response summary. Single channel high-gamma band response summary showing variations by voltage and distance for Sheep 1 (A), Sheep 2 (B), and Sheep 4 (C). Solid color lines represent intradural SC stimulation while dashed lines represent epidural stimulation. Traces are color coded according to the distance of the SC stimulator from the pial surface (color bar). The y-axis denotes High-Gamma (HG) response amplitude while the x-axis denotes the stimulation voltage (log spacing).

**Figure 5:**
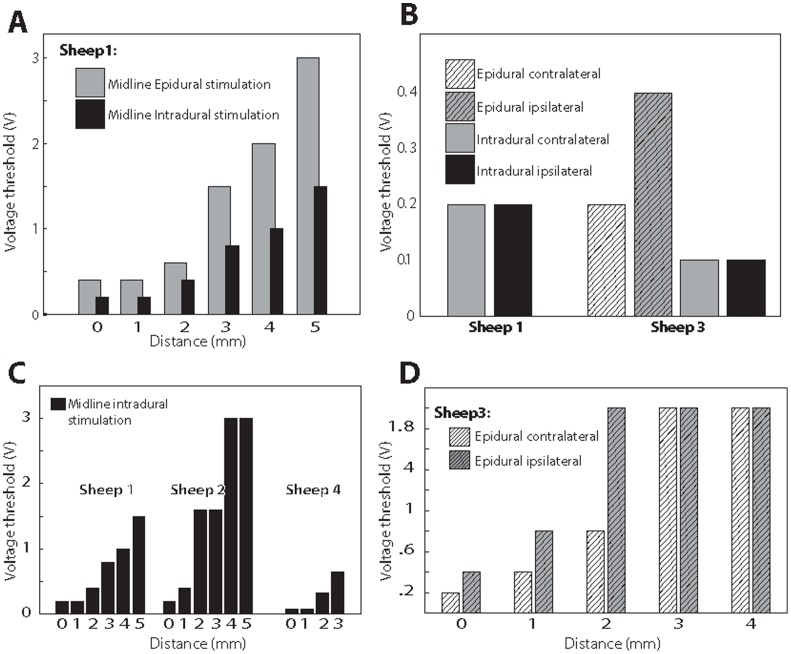
Summary of voltage thresholds needed to evoke high gamma band response (VT). (A) comparison between midline epidural stimulation and intradural stimulation. Y-axis represents VT. X-axis denotes the distance of the neurostimulator from the dorsal surface of the spinal cord. (B) Comparison between Contralateral and ipsilateral spinal cord stimulation for intra- and epidural stimulation. (C) summary of VT (y-axis) using spinal electrical stimulation of the midline in three sheep. X-axis represents the distance of the neurstimulator from the midline. (D) Comparing VT between ipsilateral and contralateral dorsal column electrical stimulation at multiple distances (X-axis).

**Figure 6:**
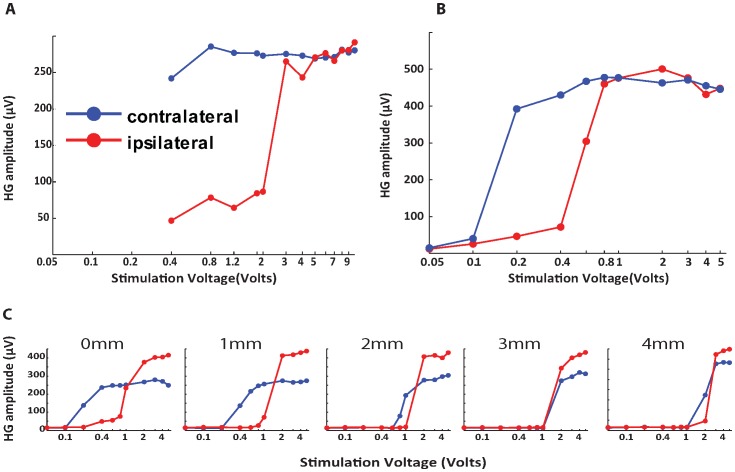
Ipsilateral Versus contralateral spinal cord stimulation. High gamma response recorded from a subdural grid placed on the right cerebral hemisphere plotted as a function of spinal cord stimulation voltage (scale is log10 separated). The red trace denotes ipsilateral SCS (right-sided stimulation) and the blue trace denotes contralateral SCS (left-sided stimulation) in Sheep 1 intradural series (A), Sheep 4 intradural series (B), and Sheep 4 epidural series (C). The numbers above the dotted line in C denote the distance in millimeters of the neurostimulating probe from the dorsal aspect of the spinal dura mater.

Evoked potentials and high-gamma responses were consistently observed over the same subdural grid contacts in all blocks. There was no striking difference between intra- and epidural stimulation in terms of evoked potential waveform shapes and topographic distributions (Figure 3A). This similarity between epidural and intradural stimulation effects was also seen in the high-gamma band (brain oscillations between 75 and 150 Hz) responses (Figure 3B). Noticeably, as distance increased higher voltages were required to evoke a detectable cortical response. It was also evident that as SC stimulation voltage increased, the magnitude of high-gamma band responses as a function of stimulation intensity increased gradually in a sigmoidal fashion (Figure 4A). As with epidural stimulation, intradural stimulation showed a similar association between high-gamma band responses and increasing voltage. Midline intradural stimulation achieved a significantly higher plateau value (t-test; p<0.05) of high gamma-response: 289.1 µV was the mean for channel 33 (276.3–301.9 µV for the 95% CI) for distances between 0 and 5 mm, when compared to midline epidural stimulation at the same distances, for which 226.7 was the mean for channel 33 (201.9–251.5 µV for the 95% CI) (Figure 4B). The voltage threshold required to detect high-gamma band oscillations was less with intradural stimulation than with epidural SC stimulation for all distances (Figure 5). When comparing left and right intradural SC stimulation, some degree of evoked cortical activity was observed with both stimulus conditions at the lowest voltage setting, as per Figure 6. Yet, the shape of the curves in Figure 6A indicates that the amplitudes of the cortical responses were, under almost all stimulus conditions, larger for contralateral spinal stimulation compared to ipsilateral spinal cord stimulation.

Sheep2: Intradural stimulation was performed at 6 distances from the dorsal midline (0, 1, 2, 3, 4, and 5 mm) as well as 14 voltages per distance (0.2, 0.4, 0.8, 1.2, 1.6, 2, 3, 4, 5, 6, 7, 8, 9, and 10 V). The total number of blocks was 84, each taking two minutes to complete. The distance-voltage response curve (Figure 4B) was consistent with that of sheep 1 (Figure 4A). When the neurostimulator was placed on the SC’s pial surface (0 mm distance), the threshold for observing a visible evoked response was 0.2 V. When the stimulator was placed 5 mm away from the dorsal surface, the threshold was 3 V (Figure 5). The mean voltage threshold required to begin inducing high gamma responses across all the distances tested was 1.6 V, with a 95% CI of 0.6–2.6 V (see the rising portions of Figure 4b). In the channel showing the highest evoked response magnitude (channel 51), the mean of the high-gamma band plateaus was at 116 µV, with a 95% CI of 114–118 µV (see the upper flat portions of Figure 4B).

Sheep 3: The third animal underwent epidural SC stimulation 2 mm lateral to the midline (left and right), at multiple distances for each lateral position (0, 1, 2, 3, and 4 mm), and 11 voltages per distance: 0.05, 0.1, 0.2, 0.4, 0.6, 0.8, 1, 2, 3, 4, and 5 V (Figure 6C). Subdural stimulation was also carried out at 2 mm lateral to the SC midline (left and right) at a single distance (0 mm) using 11 voltages: 0.05, 0.1, 0.2, 0.4, 0.6, 0.8, 1, 2, 3, 4, and 5 V (Figure 6B). The total number of blocks was 68, each taking two minutes to complete. The association between distance and voltage was observed to be similar to that of the previous two sheep. As the voltage was increased, the SSEP magnitude and high-gamma band response increased in a sigmoidal fashion. Despite starting at the same voltage threshold, 0.1 V, left-sided (contralateral to grid) intradural stimulation reached a plateau value at a lower stimulus voltage when compared to right intradural stimulation, given its steeper ascent of high-gamma band response when stimulation voltage was increased (Figure 5B). This effect was seen more clearly with left versus right epidural stimulation since the voltage thresholds were lower when the epidural stimulator was at 0, 1, and 2 mm away from the cord (Figure 5). When comparing plateau values, intradural stimulation established a larger high-gamma plateau of 383 µV (with a 95% CI of 371–395 µV) when compared to epidural stimulation which had a mean of 279 µV (with a 95% CI of 260–298 µV). When making comparisons at the same distance (0 mm), the intradural SCS voltage threshold was lower than the epidural threshold for both left (0.1 and 0.2 V for intra- and epidural) and right (0.1 and 0.4 V for intra and epidural) stimulation (Figure 5). This finding was consistent with results seen in the first animal.

Sheep 4: The fourth animal underwent only midline intradural stimulation at 4 distances from the dorsal surface of the SC (0, 1, 2, and 3 mm) and at eight voltages per distance (0.02, 0.04, 0.08, 0.16, 0.32, 0.64, 1.28, and 2.56 V). The total number of blocks was 32, each taking two minutes to complete. The finer voltage range was used to enable a more precise detection of stimulation thresholds for intradural stimulation. As predicted, a voltage-to-high-gamma band response behavior was observed and found (Figure 4C) to behave similarly to those in the previous experiments. The voltage thresholds needed to induce an observable high gamma response were 0.08, 0.08, 0.32, and 0.64 V for distances of 0, 1, 2, and 3 mm away from the pial surface (Figure 5).

## Discussion

### Assessment of the Findings

The current experiments were carried out to provide information useful in the design and development of a new direct spinal cord stimulation system. Computer modeling methods have been used extensively to examine how spatial relationships between stimulus delivery current sources and the spinal cord affect patterns of activation of neural elements within the spinal cord. We performed *in vivo* studies in order to objectively test certain assumptions inherent in these models, and to provide empiric data regarding electrophysiological activation thresholds for direct spinal cord stimulation. SSEPs recorded from a surface ECoG grid provided a reliable and objective measure of cortical activity evoked by electrical stimulation of the spinal cord.

To achieve these objectives we systematically studied cortical evoked potential and high gamma magnitude differences while changing the position of the stimulating leads relative to the surface of the spinal cord. We also examined the effects of having an intact dural membrane between the stimulating electrode and spinal cord by conducting experiments before and after a durotomy procedure (Figure 1A). By placing the stimulator in different locations (epidural vs. intradural; midline vs. lateral) and at different distances away from the cord, we addressed several key questions. Does intradural, direct SC stimulation evoke brain responses that are qualitatively similar to those observed when stimuli are delivered epidurally? What are the voltage thresholds for electrophysiological activation of spinal cord pathways when the neurostimulator is placed in different positions relative to the spinal cord? How do local brain responses change across the recording grid as we change these parameters (voltage and positions)? These questions address fundamental issues that are critically important to the design of spinal cord stimulation systems.

First, we demonstrated that intradural SCS induces a cortical response similar to that observed with epidural stimulation. All current clinical spinal cord stimulators have leads positioned in the epidural space. This similarity in brain responses following epidural and intradural spinal cord stimulation was manifest both in the shape as well as topographic distribution of evoked potentials and high gamma oscillations recorded from the subdural grid placed on the sheep’s cerebral cortex (Figure 2). The next step was to examine the change in high-gamma band response amplitude as a function of the distance separating the stimulating electrode from the spinal cord surface, and the applied voltage. By varying the stimulus voltage between 0.05 and 18 V, and the neurostimulator distance from the cord over the range from 0 to 10 mm above the surface, we were able to deduce voltage-distance-response relationships for every recording channel. As anticipated, and in keeping with previous computer modeling reports, a consistent change in the high-gamma band with distance and applied voltage was observed over all sheep. With increasing voltage, the high-gamma band magnitude increased steadily and nonlinearly in a sigmoidal manner. The plateau cortical response value was higher and required a lower voltage threshold when the stimulator was placed intradurally as compared to epidurally. At the same time, distance was a substantial factor since stimulation thresholds were lowest and high gamma responses were strongest when the stimulating electrodes were closest to the SC (0 mm distance). This response decreased gradually as the distance from the cord surface increased.

From the data for animals 1 and 3, we concluded that stimulating the SC with the dura intact necessitated higher voltage thresholds (about 2x) to evoke detectable high gamma-band responses in the brain when compared to the post-durotomy condition (Figure 5). We also observed that the maximum high gamma response (i.e., the plateau value) of epidural stimulation was less than the plateau value with intradural stimulation (Figures 3A, 6B, 6C). These findings suggest that the presence of dura between the stimulating electrode and spinal cord serves to attenuate to some degree the stimulus signal reaching the spinal cord. For intra-dural, direct SC stimulation the voltage-response curve had a steeper ascent to plateau with contra-lateral SC stimulation when compared to ipsi-lateral SC stimulation (Figures 6A, 6B). This effect of medial-lateral position relative to the mid-line was also seen with epidural placement of the stimulator (Figure 6C). For epidural stimulation, not only did left-sided (contralateral) spinal cord stimulation result in a cortical response pattern having a steeper ascent, but it was clear that the voltage thresholds for evoking a response were lower at close stimulation distances from the pial surface (0, 1 and 2 mm, Figure 5). These findings demonstrate the importance of stimulus source location relative to the spinal cord when functionally activating dorsal column axons. Not only did we find differences between epidural and intradural stimulation, but stimulating the contralateral dorsal column is more effective in evoking cortical potentials and high gamma oscillations than ipsilateral stimulation.

### Potential Clinical Implications

It is estimated that more than 35,000 SCS devices are surgically implanted for a range of clinical conditions each year in North America alone [20]. Yet, our full understanding of the underlying therapeutic mechanism is still unfolding. As part of the procedure, stimulator leads are placed in the epidural space with the intent of delivering neurostimulation to the underlying dorsal columns. In order to have a physiologic effect in attenuating pain, the electric pulse from the stimulator lead has to propagate through the dura matter, a layer of CSF surrounding the cord, and the pial layer to finally reach the dorsal column axons. Generating currents that penetrate all these barriers prior to reaching the dorsal column axons and at the same time retain target specificity constitutes a fundamental limitation to the epidural approach. It is estimated that only 10% of stimulation current delivered from the epidural space reaches the SC surface, and the depth of effective penetration below the pial surface is only 200 to 250 um [11]. This has been largely attributed to the shunting effect of CSF due to its high conductivity, 1.8 S/m, which is one of the highest in the body [21]. As a result, the stimulators currently in use are unable to functionally modulate many potential targets within the human spinal cord that might be effective in alleviating pain symptoms. Moreover, shifts in lead position over time decrease the coupling strength relative to the independently moving and pulsating cord. All of these factors make it very difficult to activate more than just a thin layer of sub-surface fibers abutting the dorsal pial surface of the spinal cord using epidural approaches.

The gate theory of pain asserts that in order to reduce transmission of pain related to a specific dermatomal segment (segment-specific pain), large diameter fibers of that same segment transmitting kinesthetic and discriminative touch information should be activated. In order to maximize the utility of the gating mechanism of pain, it would be reasonable to activate the maximal sum of segment-specific large diameter axons while sparing nearby pain fibers entering the cord from the dorsal rootlets and other non-targeted sensory pathways. In other words, it would be best if the stimulus currents activated only their intended targets in the dorsal columns while leakage currents going to neighboring sensory structures are minimized. One way to achieve this is by selectively activating the segment-specific axons along the medio-lateral dimension of the dorsal column [22], [23], [24] while at the same time stimulating a larger sum of sensory axons by maximizing dorso-ventral penetration of the dorsal column; preferably at the lowest stimulation voltage possible. With this approach more segment-specific axons would be recruited, and this should be associated with improved clinical efficacy. The non-myelinated small-diameter C-fibers transmitting painful stimuli have a higher excitation threshold when compared to large diameter myelinated Aβ fibers transmitting kinesthetic and discriminative touch sensations. If we plan to selectively activate those large diameter fibers and spare the C-fibers, using low stimulation intensities will be of prime importance. Given this differential excitation susceptibility, using lower voltages provides an additional advantage by further widening the therapeutic window. With that strategy, analgesic effects should be enhanced and adverse effects of stimulation induced pain and paresthesias avoided. To implement that strategy and accomplish maximal segmental pain relief, we believe that the best approach is to directly stimulate those target fibers by placing the stimulating electrode directly on the surface of the SC [12], [16], [19]. Direct spinal cord stimulation will maximize the exploitation of postulated gating mechanisms in two ways. First, by bypassing the shunting effect of CSF, it becomes feasible to deliver current to deeper layers in the dorsal columns. With less CSF shunting, direct stimulation can provide better current penetration, resulting in activation of axons located a greater distance below the pial surface. The more dorsal column axons that are activated, the greater the ‘dose effect’ achieved which, in turn, should be associated with greater pain relief. Second, with the lower voltage, requirements comes the advantage of better control of the current densities across the mediolateral expanse of the dorsal columns (segment-specific). In other words, when comparing it with epidural stimulation, direct spinal cord stimulation is capable of targeting specific dorsal column segments more effectively and with a higher degree of specificity. The lower voltage costs of placing the stimulator leads right on the cord’s surface also translate to less current leakage into neighboring neural structures, more energy efficient electronic stimulation systems and, for the first time in humans, the ability to perform direct SC recordings *in vivo* thus opening up new avenues for human spinal cord neuroscience research.

A number of limitations must be considered when interpreting the results of the present study. These experiments were designed to address specific questions about the importance of distance and relative position of a stimulation source as these variables relate to physiological activation of the spinal cord. The experiments were performed under general anesthesia in normal sheep. Therefore, the effects of electrical stimulation in this setting on pain, or the physiology of neural systems that were not studied but are implicated in the pathophysiology of pain (eg., segmental spinal cord neural circuits distal to the site of electrical stimulation), are unknown. Another important limitation is that a restricted range and number of incremental gradations of spinal cord stimulation voltage settings were used. Because of time constraints and complexities associated with obtaining recordings at multiple locations in the same experimental preparation, it was not feasible to obtain finer-grained voltage threshold determinations for each location studied. This results in some instances in artificially high threshold estimates. For example, in Figure 6A, even though it was clear that contralateral stimulation is more efficient than ipsilateral stimulation, our objective algorithm that estimates the voltage threshold V_t_ (as calculated with the expression given above) deemed that both states (ipsi- and contralateral stimulation) have equal thresholds of 0.4 V when the stimulator is placed directly on the spinal cord’s surface.

### Directions of Future Work

The results of our acute ovine study have helped to inform the design of the I-Patch implant that will be used in an upcoming series of chronic studies also to be carried out using an ovine model. The goals of that work will be to look for any intermediate to long-term (>1 month) effects caused by the continuous presence of the I-Patch on the pial surface of the spinal cord, and to assess the functional effects of direct SC stimulation using experimental protocols designed to test motor, somatosensory and bowel/bladder functions. Video gait analysis will be one of the functional tests performed. This experimental testing method was developed originally as a means of obtaining objective outcome measures following interventions designed to treat spinal cord injuries [25], [26]. We anticipate that the same methods should provide useful insights into assessing response to intradural spinal cord stimulation. At the completion of these experiments a careful post-mortem pathological assessment of the animal spinal cords will be performed. The tissue will be analyzed for evidence of inflammation, scar formation, or neurotoxicity associated with the implantation and use of the I-Patch. To minimize the potential for harmful tissue interactions, the I-Patch will be constructed of materials similar to those employed in the fabrication of its nearest predicate device, the auditory brainstem implant (ABI), which has been used safely and efficaciously in patients for decades [27], [28], [29]. Nevertheless, as part of a rigorous safety assessment, the tissue will be analyzed for any signs of electrical, mechanical or biochemical-induced damage to the spinal cord, using protocols similar to those described by Sutton [30] and Manrique *et al*. when evaluating ABI implants [31], [32]. Finally, for actual human implantation, we are developing a specially designed version of the I-Patch that will be able to accommodate spinal cord movement as the patient changes posture. The design goal for it is to keep the electrode-bearing surface of the I-Patch in continuous contact with the pial surface of the spinal cord during any kind of flexion, extension or twisting motions. This is accomplished by arranging the intradurally deployed leads of the device in a looped configuration that provides for an adequate range of device co-movement with the spinal cord, while also applying the delicate restoring force needed for it to maintain safe contact with the pial surface.

### Conclusions

In previous reports, we presented a new device concept designed to deliver electrical stimuli directly to the spinal cord, thus overcoming a key limitation common to all current epidural SCS devices: a limited capacity to functionally access neural pathways within the spinal cord. The current study, however, did not measure pain scales following spinal cord stimulation in sheep and therefore, further studies will be needed to determine if this novel device is indeed capable of overcoming the fundamental limitation of existing spinal cord stimulators for treating chronic refractory pain. In the present systematic set of experiments, the results highlighted the importance of stimulating electrode positioning relative to the spinal cord surface in evoking cortical responses in sheep. In particular, we have characterized the effect of stimulus magnitude and stimulus source location relative to the spinal cord on cortical high-gamma band responses within sheep sensory cortex. The voltage needed to evoke responses to direct stimulation of the spinal cord surface is as low as 0.2–0.4 V (95% CI). Those responses quickly became stronger and reached plateau levels as the applied voltage increased. We also compared activation thresholds of different stimulation positions at different distances and medio-lateral locations above the dorsal surface of the spinal cord. We demonstrated that the intact dura mater alters stimulation requirements, necessitating higher voltage thresholds and evoking a weaker maximal high gamma response when compared to intradural stimulation, even when the distances between the stimulating electrode and the spinal cord are the same for the two conditions. We have shown that important factors affecting voltage thresholds to evoke a somatosensory evoked response and an increase in localized high frequency cortical oscillations include the presence of the dura mater, the distance of the probe from the underlying cord surface, and the position of the stimulating contacts relative to the midline plane. These findings are consistent with those predicted in earlier theoretical computer stimulation modeling studies. Given the steep voltage-response curves and the dependence of cortical response on the location of the neurostimulator, fine stimulus intensity control as well as fine spatial coverage are essential and critical factors to consider in the design of spinal cord stimulation systems.
